# Day-Length Is Involved in Flooding Tolerance Response in Wild Type and Variant Genotypes of Rootstock *Prunus cerasifera* L.

**DOI:** 10.3389/fpls.2019.00546

**Published:** 2019-05-03

**Authors:** Calogero Iacona, Laura Pistelli, Marco Cirilli, Lorenzo Gatti, Roberto Mancinelli, Maria Nicolina Ripa, Rosario Muleo

**Affiliations:** ^1^Department of Agriculture, Food and Environment, University of Pisa, Pisa, Italy; ^2^Laboratory of Molecular Ecophysiology of Woody Plant, Department of Agricultural and Forestry Sciences, University of Tuscia, Viterbo, Italy; ^3^Department of Agricultural and Environmental Sciences, University of Milan, Milan, Italy; ^4^Tree and Timber Institute, National Research Council of Italy, Sesto Fiorentino, Italy

**Keywords:** anoxia, off-season flooding, photoperiod, physiological plasticity, plum, seasonal changes, waterlogging adaptation

## Abstract

Current and predicted climate changes scenarios require crops with an improved adaptability to mutable environmental features, such as, hypoxia for the root system. In order to overcome the reduction of oxygen, plants activate coping mechanisms and strategies. *Prunus* spp. are hypoxia-sensitive woody species and although many information has been gathered over the last decades, many physiological mechanisms remain unclear. To verify whether anoxic plant responses are also regulated by photoperiod, plants of Mr.S.2/5-*WT* plum, and its variant genotypes *S.4* tolerant (plus) and *S.1* sensitive (minus) to flooding, were grown in a greenhouse and were submitted to natural photoperiod (NP) and to constant photoperiod (CP) from mid-July until the first 10 days of October. From mid-September plants from each genotype, grown under the two photoperiods, were divided into two groups, and one of them underwent long-term flooding. Gas exchange parameters, energetic and biochemical activities, leaf chlorophyll contents, and stress symptoms were measured at different times, whereas soluble sugars were quantified in leaves and roots 14 days after flooding, when stress symptoms in *WT* and *S.1* became prominent. Seasonal changes in the photoperiod played a role in the adaptability to anoxia, although flooding stress response differed among the three genotypes. Anoxia affected leaf gas exchange and *S.4* flooded-leaves retained higher ACO_2_ under conditions of NP and CP. Leaf soluble sugar concentration differed among genotypes. Regardless the photoperiod, *S.4* anoxic-leaf sugar concentration was the lowest, except for sorbitol. *S.4* anoxic-roots under CP accumulated the highest levels of sucrose and sorbitol. Influences of the photoperiod were observed in *WT* and *S.1* anoxic-leaves, whereas *S.1* anoxic roots accumulated the lowest concentration of sugars, regardless of photoperiod. Leaf and root respiratory activity in flooded-plants was highest in *S.4*, and ADH activity increased in all flooded plants under CP but the highest activity was observed only in *S.1* under NP during flooding. Results are consistent with the hypothesis that the *S.4* genotype has a plastic adaptability to flooding stress, escaping from the photoperiod regulatory cross-talk system, and can better cope with the new scenarios generated by climate changes.

## Introduction

Climate change are causing seasonal changes in air temperatures and local rainfall distribution, and more frequent extreme precipitations events can occur (IPCC, 2014). These events are mainly concentrated in spring and late summer and they damage the ecosystem conservation and agricultural production, affected by soil waterlogging (Alpert et al., 2002; McFarlane and Williamson, 2002; Kijne, 2006). The gas emission from the soil due to water excess, coupled with the low availability of oxygen (hypoxia) and/or complete O_2_ depletion in the rhizosphere, generate inhospitable condition of flooding, and cause the reduction in growth and crop productivity for a wide range of plants (Wang and Jiang, 2007; Arbona et al., 2008; Voesenek and Bailey-Serres, 2013). Without oxygen, plants shift in few hours from aerobic to anaerobic respiration undergoing ethanol fermentation, it regenerates NAD^+^ via alcohol dehydrogenase (ADH), meanwhile the ATP yield is strongly reduced (Bailey-Serres and Voesenek, 2008). Under anaerobic respiration the anoxic cell could increase its rate of glycolysis to equate the energy levels of aerobic cells (Greenway and Gibbs, 2003). Flooding stress, a few days after its onset, is frequently accompanied by a reduction and/or complete blockage of photosynthesis and gas exchanges, thus reducing carbohydrate-availability to roots or other storage plant organs (Núñez-Elisea et al., 1999; García-Sánchez et al., 2007). Long-term exposure to flooding also induces a decline in leaf and stem water content causing accumulation of leaf carbohydrate, epinasty, leaf desiccation with premature senescence and abscission (Kozlowski, 1997; McLeod et al., 1999; Pezeshki, 2001). These events are preceded by chlorophyll damage due to the impairment of Photosystem II (PSII) and light harvesting complexes (LHCII), with the consequent formation of oxidant compounds, which in turn damage cellular components, lipids and proteins, resulting in a decrease in ATP and NADPH production especially in the chloroplast, necessary for carbon fixation reactions (Cai and Xu, 2002; Mauchamp and Méthy, 2004).

Plants perceive the flooding stress differently, showing intensity, timing and duration peculiar for each species; tolerance involves the activation of physiological complexes, metabolic pathways and molecular networks (Bailey-Serres and Voesenek, 2008; Pucciariello et al., 2014; Rubio-Cabetas et al., 2018; Zhu et al., 2018). The set of various morphological and physiological adaptations confers flood tolerance to woody plants. Major adaptations include the induction of adventitious roots to compensate the decay of portions of the original root system, the formation of lenticels and aerenchyma tissues in roots and stems to facilitate O_2_ and CO_2_ movement (Kozlowski and Pallardy, 2002).

Temperate trees subject to flood are simultaneously exposed to other environmental conditions, such as the seasonal variation of temperature and photoperiod. Trees enter a state of dormancy during the process of vernalization and this allows them to overcome the adverse winter condition, through adaptive mechanisms that protect flower buds (Preston and Sandve, 2013). Therefore, besides being an important trait for plant adaptation, vernalization has also a great agronomical importance (Iqbal et al., 2007). Dormancy or quiescence in perennial plants is controlled by temperature and daylength (photoperiod) seasonal changes (Campoy et al., 2011). Although the photoperiod is a reliable and predictable environmental cue at middle and higher latitudes, however, its role in the onset of dormancy in some Rosaceae species is strongly debated (Heide and Prestrud, 2005; Cooke et al., 2012). For *Prunus* species, vernalization starts from mid-July and lasts throughout the summer; it is stimulated by day-shortening, frequently coupled with the first reduction of temperatures, that trigger to arrest the aerial plant growth, and to set terminal bud as well as wintering cold acclimation processes (Looney and Jackson, 2011). In recent years, researchers highlighted some molecular mechanisms regulating the wintering processes, suggesting that photoperiod enables plants to program for periodic future events (Bouché et al., 2017). Photoperiod, through the circadian rhythm depending on an internal clock system, synchronizes the internal rhythm with the environment, a crucial aspect for plant adaption to adverse conditions (Seo and Más, 2015). Investigations have been undertaken to understand the cross talk between the circadian clock and abiotic stresses (e.g., drought, cold, heat, osmotic) in the *Arabidopsis* model plant (Grundy et al., 2015) and in trees, and on the relationship existing between circadian clock and bud dormancy induced by cold (Artlip et al., 2013; Lloret et al., 2018). However, there are no known reports on the relationship with prolonged submergence during off-season waterlogging. Through dormancy, woody plants become tolerant to the adverse environmental conditions of autumn and winter, like waterlogging due to rainfalls. So far, literature is scarce on cross-talk between the regulatory signaling of photoperiod and plant flooding response; the accepted results deal with plant-water relationship and stomatal conductance as influenced by flooding stress imposed at the beginning and at the end of the photoperiod (Dell’Amico et al., 2001; Else et al., 2009).

*Prunus* species are reported to be intolerant to flooded conditions and it has been observed that they decline or die under excessive waterlogging (Ranney, 1994; Rubio-Cabetas et al., 2011; Amador et al., 2012; Pistelli et al., 2012; Almada et al., 2013; Rubio-Cabetas et al., 2018). This research aims to establish whether the adaptive response to long-term flooding is linked to a photoperiodic regulatory system of plant phenology in the wild type (*WT*) of rootstock Mr.S. 2/5 of *P. cerasifera* and in two variants, with divergent tolerance (Pistelli et al., 2012). In other words, whether the different adaption behaviors observed among the genotypes should be also associated to mutations in the photoperiodic responses of plants generating circadian entrainment to the natural light-dark cycle across seasons.

## Materials and Methods

### Plant Materials and Experimental Stress Conditions

Five months old plantlets of the Mr.S.2/5 *WT* (*P. cerasifera* Ehrh), *S.1* (minus variant) and *S.4* (plus variant) genotypes (Muleo et al., 2006) were acclimatized in a greenhouse to *extra-vitro* conditions at University of Tuscia (DAFNE-department, at the latitude of 42° 42′N), and cultured in pots containing 1 L of soil composed of 45% clay, 45% sand and 10% silt.

To evaluate photoperiod-mediated response to flooding, 20 days after the summer solstice, the plants (approximately 15 cm long) were randomly divided into two groups: 40 plants of each clone were grown under Natural Photoperiod condition (NP) and other 40 potted plants of each clone were grown under Constant Photoperiod condition (CP), and maintained until October, after the soil waterlogging experiments. The trials were repeated in 2015 and 2016 for 2 years consecutive tests. CP was obtained by keeping the time of lighting similar to summer solstice (16:8 h light:dark). It was provided by fluorescent lamps (TLD18W/33 cool-white fluorescent tubes), emitting 6 μmol m^-2^ s^-1^ measured just above the height of the plants, and turned on before dusk. During the summer, the plants were exposed to a maximum PAR (LI-170; LICOR Inc., Lincoln, NE) of 1500 μmol m^2^ s^-1^. Average day/night temperature was 26°/18,6°C, and the daily cycle of relative humidity ranged between 35 and 95% during the summer period. Plants were regularly ferti-irrigated three times a week with 100 mL of half-strength Hoagland’s solution.

In mid-September, the 40 plants of each genotype grown under each photoperiod were randomly divided into two groups. Twenty uniform plants were exposed to complete soil submersion for 28 days by placing them in large plastic containers (90 cm × 60 cm × 25cm) in a randomized pattern, filled 3 cm above the soil surface with tap water. The other 20 plants were kept under well-drained soil (normoxic plants) and watered every day. The flooding stress experiment was performed in 2 years consecutive tests. The experimental pattern was a 3 × 2 × 2 factorial of plant genotypes (3) × photoperiod (2) × stress (2, control and flooding), with 16 replicate plants completely randomized in each treatment. The flooding experiments were carried out in a greenhouse at a daily cycle of relative humidity between 40 and 100%, at the day/night average temperature of 22.1°/17.6°C and daily vapor pressure deficit of 4–10.2 kPa and at a maximum photosynthetic active radiation of 1240 μmol m^-2^ s^-1^. During the experiment, oxygen depletion measurements were periodically recorded with a portable dissolved oxygen meter (Hanna Instruments, Lansing, MI, United States) inserted into the soil at 5 cm depth. The diffusion of O_2_ in the soil of non-flooded plants was always constant (903.3 ± 40.4 ppm).

### Plant Growth and Morphological Adaptation

In all the trials, the plants height, from the soil surface to the top of the apical bud, and the amount of phytomers, were measured the day before the soil submersion treatments (Day 0) and at the end of the photoperiod treatment and flooding test (28th day). Abscised and/or dried leaves were evaluated on Days 0, 7, 14, 21, 28 of the soil waterlogging experiments. Leaf and root morphology, and hydration status were also monitored at the end of the test. The dry weight of leaf and root samples was obtained by oven-drying plant material at 70°C to a constant weight.

### Leaf Gas Exchange

Gas exchange parameters were measured in the 1st and 4th healthy, full expanded mature leaves, when available, during the 2016 test period. Measurements were conducted at the 7th and 14th day, because the *S.1*- and *WT*-plants up to that period retained the physiological activity on most of the surface of leaf lamina. Leaf measurements of net CO_2_ assimilation (A_CO2_, μmol m^-2^ s^-1^), stomatal conductance (*gs*, mmol H_2_O m^-2^ s^-1^), intercellular CO_2_ concentration (C_i_, μmol m^-2^ s^-1^) and transpiration rate (E, mmol H_2_O m^-2^ s^-1^), were performed at the end of the experiment using a portable infrared gas analyzer with a leaf chamber (LI-6400XT, LI-COR Inc., United States), with a 0.25 l cuvette. The LICOR-6200 was equipped with an external light source (Model QB1205LI-670, Quantum Devices Inc. Barneveld, WI, United States) to maintain a constant PAR of 600 μmol m^-2^ s^-1^ during measurements. All measurements were carried out in the morning from 8 to 10 a.m. to avoid high temperatures, on five plants per clone and per treatment. During all measurements, leaf temperature was 26 ± 1°C and leaf-to-leaf-to-air vapor pressure difference was 2.4 ± 0.4 kPa within the cuvette.

### Chlorophyll Determination

Chlorophylls were determined in leaves of each genotype at the end of trials (28th day) from two diameter leaf discs (Ø 0.6 cm), sampled from the mid-lamina area of the intervene zone. Chlorophyll *a* and *b* were extracted and quantified according to Moran (1982).

### TTC Reactivity Test

The triphenyltetrazolium chloride (TTC) reactivity test was used to measure tissue vitality and respiratory activity of leaves and roots. Analyses were carried out on 100 mg of fresh tissues, using the same protocol as described in Pistelli et al. (2012). The reactivity of the samples with TTC was measured as absorption of triphenyl formazan per g dry mass (A_520_ g D.W.^-1^).

### Soluble Sugars Analysis

Extraction and quantification of soluble sugars (sorbitol, sucrose, glucose, and fructose) were performed by sampling fully expanded leaves and roots of plants from all genotypes. Measurements have been conducted at 14th day in control and flooded-plants. The amounts of glucose, fructose and sucrose were then determined using a coupled enzymatic assay method (Pistelli et al., 2012). The efficiency of the methods was tested using known amounts of carbohydrate (glucose) as standards. Sorbitol determination was carried out using spectrophotometric analysis using La Roche Kit, monitoring the formazan formation at 492 nm (Bergmeyer et al., 1974). Carbohydrate concentrations were expressed in units of mg g^-1^ DW.

### ADH Determination and Total Protein Determination

Root samples (at 0, 21st, and 28th day of treatment) were gently washed in water, and the main root apex (the last 3 cm from the tip) was cut with a razor blade and rapidly frozen in liquid nitrogen. Protein extraction and ADH activity were carried out as already described (Pistelli et al., 2012). Total protein contents were determined according to the method of Bradford (1976) by using bovine serum albumin (BSA) as standard.

### Statistical Analysis

The data were subject to variance analysis by using a three-way ANOVA test, performed by the SigmaStat 3.1 package (SYSTAT software Inc., Chicago, IL, United States). Effects of clones, photoperiod and stress treatment on leaf gas exchange, chlorophyll amount and TTC activity were evaluated. When treatment interaction terms were significant (*P* < 0.05), treatment means were separated using Tukey’s multiple range test. The statistical samples for leaf gas exchange, chlorophyll amount and TTC activity included five plants, while for the other biochemical parameters three biological independent repeats were used. Percentage data before the ANOVA test were transformed in arcsin values before analysis in order to homogenize the variance and the data shown in the results were back-transformed. Differences were accepted as statistically significant when *P* < 0.05. PCA (principal component analysis) and MDA (Multigroup Discriminant Analysis) factorial discriminant analysis were carried out on morpho-physiological traits and carbohydrates accumulation. MDA and PCA allowed us to discriminate among genotypes under anoxia and photoperiod the changes of the morpho-biochemical traits in response to the stress. All analyses were performed in JMP 4.0 statistical software package (SAS Institute Inc., Cary, NC, United States).

## Results

### Anoxic Status

The hypoxic condition during the flooding treatment has been determined by the diffusion of O_2_ in the soil. In the soil of normoxic exposed plants, the values were always constant (932.4 ± 50.3 ppm), while in the waterlogged soil the O_2_ decreased rapidly. After 3 days the values ranged from 236.7 to 264.8 ppm and after the sixth day in the waterlogged soil anoxic conditions the O_2_ level in the waterlogging soil was maintained in the range of 10.5–29 ppm.

### Plant Growth and Morphological Response

**FIGURE 1:**
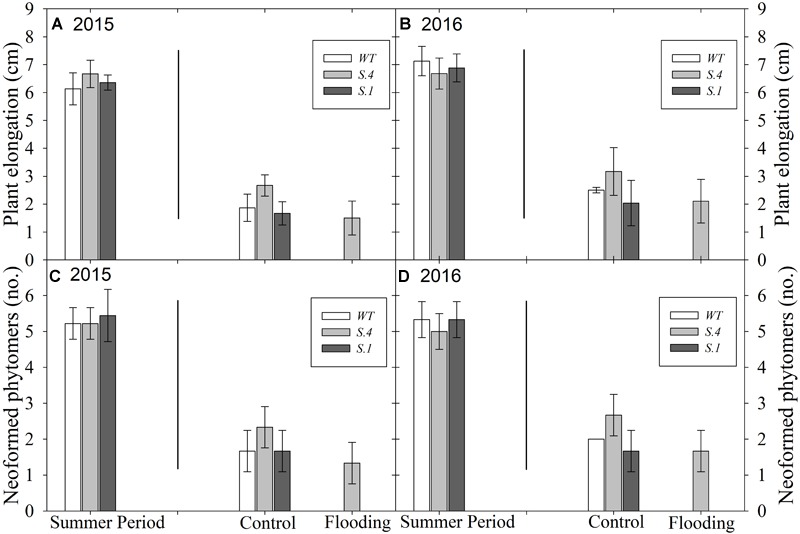
Plant growth and development under Constant Photoperiod (CP) performed in 2 years consecutive tests. In **(A,B)** is shown the plant growth detected as stem elongation; in **(C,D)** is shown the development of new laterals buds (phytomers). Lines inside the graphics separate, on the left, the plant behavior under CP starting from 10th day of July until the beginning of flooding treatment, and on the right, the plant behavior under flooding treatment starting from the 10th day of September. All data were detected at the end of waterlogging treatment (*n* = 5 biological replicate samples, ± SD).

**FIGURE 2:**
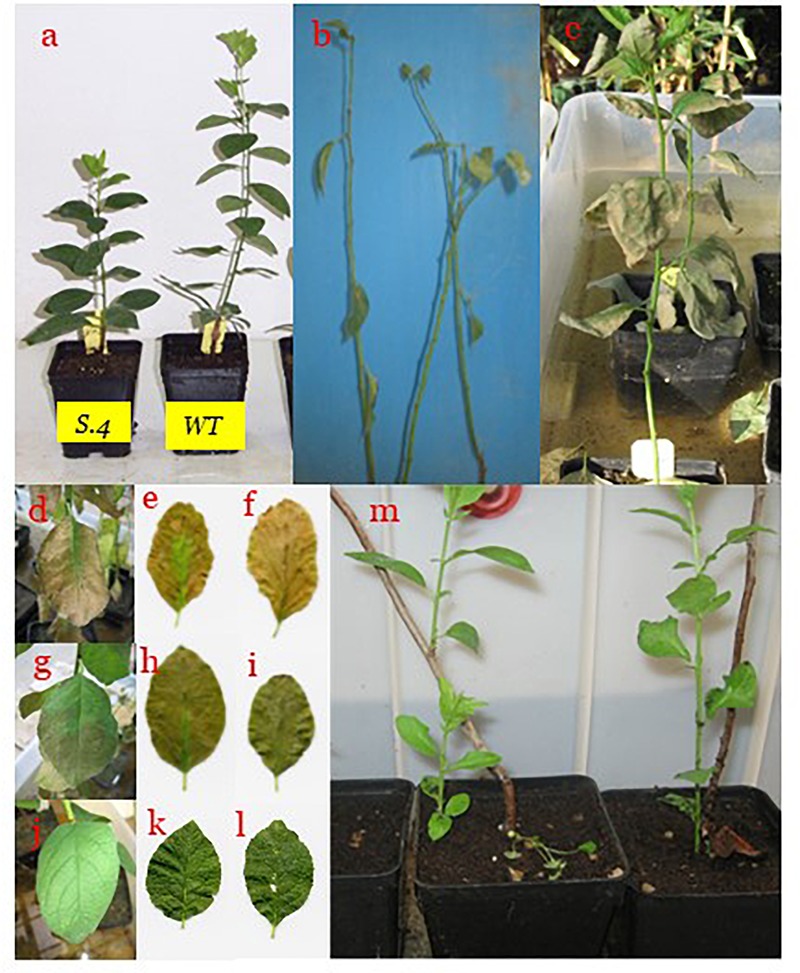
Plant behavior during treatments. Re-growth of apical bud **(A)** in *S.4* and *WT* plants exposed to the constant photoperiod (CP) since the mid-July under normoxic conditions. *S.1*-plants behaviors at the end of flooding period (28 days) exposed to CP **(B)** and to NP **(C)**. State of leaf detected during flooding (14th, 21st, 28th day) in *S.1* **(D–F)**, *WT* **(G–I)**, and *S.4* **(J–L)**, respectively. *S.4*-stressed plants exposed to CP showed loss of basal leaves, development of sprouts and suckers from lateral buds and root systems **(M)**, respectively.

Plants kept under natural photoperiod (NP) have ceased to grow and set the terminal bud immediately after the middle of July; meanwhile, the plants kept under CP exhibited stem elongation (Figure 1A,B, 2A) and development of new phytomers (Figure 1C,D, 2A), in the 2 years consecutive tests that were performed. When exposed to prolonged waterlogging, from 10th of September, plants blocked their growth, except for the plants of *S.4* (Figure 1). This genotype lost the basal leaves but developed sprouts and suckers from the lateral buds and root systems (Figure 2M).

**FIGURE 3:**
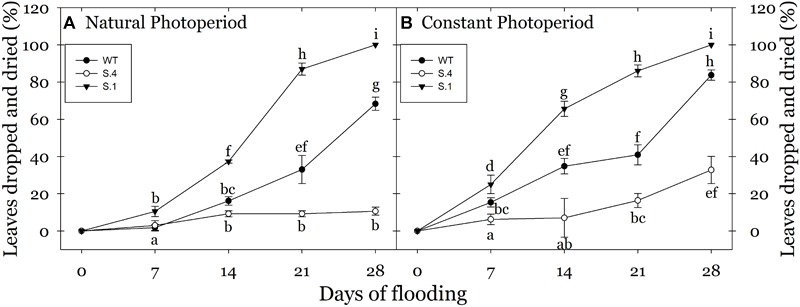
Percentage of leaves (dried or dropped) detected in plant during the flooding stress grown under NP **(A)** and CP **(B).** No abscission events have been detected in plant grown under normoxic condition (*n* = 5 biological replicate samples, ± SD). Different letters indicate statistical significance for *P* < 0.001. In the case of low value of SD, the bar is covered by the symbol.

At the end of the flooding period, the leaves of *S.1*-stressed plants were almost lost and/or had a visible loss of vitality (Figure 2B,C). The most important effect under prolonged flooding stress is the typically induction of an intense leaf epinasty, that became particularly evident after the second week of stress in *WT* plants, and in *S.1* plants since the first week of stress under CP. Extending the stress, the browning increased in the leaf area and the whole organ dried (Figure 2E,F). As a consequence, a higher percentage of epinasty in the leaves with or without consequent dropping of foliage, occurred in these plants at the end of flooding treatment (Figure 3). *WT*-plants showed a reduced response compared to that of *S.1*-plants, i.e., yellowing of the leaves after the initial days of stress treatment (Figure 2G–I, 3). Apical leaves of *WT* and *S.1* genotypes did not grow properly and at 14th day, they appeared greatly stressed with a dried large region in *S.1* (Figure 2D) or partially browning of the leaf edge in *WT* (Figure 2G).

The effect of photoperiod and its interaction with the genotype become particularly evident when comparing the percentages of dropped and dried leaves during flooding period under CP and NP conditions. CP-plants of *S.1* showed an early stress damage, with withered leaves and strong epinasty since 7th day of stress (Figure 3). At 14th day of flooding NP-plants of both *WT* and S.1 genotypes had a lower percentage of dropped leaves compared to CP-plants (40 vs. 70 and 20 vs. 40%, respectively). On the other hand, at 28 days of flooding, *S.4*-plants showed a better fitness under stress (Figure 2J–L), with absence of epinasty, lack of yellowing and browning and reduced dieback and dropping leaves (Figure 3). Although S.4-plantsunder CP showed an increment of percentage of abscised leaves compared to that under NP (35 vs. 10%, respectively), the percentage of abscised leaves was lower than *WT* and *S.1*-plants (85 and 100%, respectively). Adventitious roots only developed from the flooded *S.4*-plants under a condition of CP, and an average of 3.2 roots per plant was detected (data not shown).

### Chlorophyll Content

**FIGURE 4:**
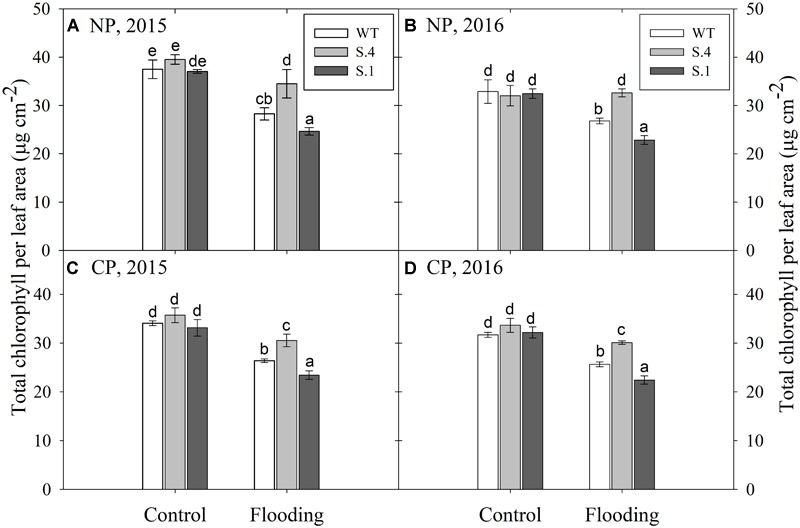
Total chlorophyll content detected on adult expanded leaves at the end of flooding experiments (28th) in plant grown under NP **(A,B)** and CP **(C,D)**, for 2 years consecutive tests. Histograms represent the average of five biological replication and the bars ± S.D. Different letters within the same year indicate statistical significance for *P* < 0.001.

The chlorophyll content was detected in adult expanded leaves located near the apex (Figure 4). The amount of total chlorophyll (per cm^2^ of leaf area) was lower in all the genotypes under CP for the 2 years tests, even if it wasn’t statistically different from NP (Figure 4 and Table 1). A similar trend has been identified during flooding conditions, although only the genotype per flooding interaction was significant (e.g., under flooding the total chlorophyll content differed among genotypes) (Figure 4). The amount of total chlorophylls was lower in *WT* and *S.1*-plants in both experimental years regardless of the photoperiod. This change was due to the reduction in the synthesis and accumulation of chlorophyll *a*, since the chlorophyll *b* was only slightly reduced in *WT* and *S.1*-plants, but it was increased in *S.4*-plants, independently of the photoperiodic regime (Table 1). Therefore, the *a*/*b* chlorophyll ratio tends to decrease under flooding conditions but the observed decrease due to the photoperiod was not significant.

**Table 1:** Level (μg cm^-2^) of chlorophyll *a, b*, and *a*/*b* ratio in control, flooded-stressed Mr.S. 2/5-*WT*, and *S.1* and *S.4* plants at the end of the flooding experiment.

Main factors		Chlorophyll *a*	Chlorophyll *b*	a/b ratio
Genotype	*WT*	20.50 b	8.75 a	2.36 b
	*S.4*	21.29 c	10.80 b	2.01 a
	*S.1*	18.50 a	8.98 a	2.06 a
Photoperiod	NP	20.55 b	9.38	2.22
	CP	19.64 a	9.63	2.07
Treatment	Control	22.75 b	9.72	2.38 b
	Flooding	17.44 a	9.29	1.90 a
Interaction			
	*WT* × NP × Control	23.69 de	9.18	2.66
	NP × Flooding	18.21 b	8.60	2.12
	CP × Control	22.66 cde	9.03	2.52
	CP × Flooding	17.46 b	8.18	2.14
	*S.4* × NP × Control	22.40 cd	9.63	2.36
	NP × Flooding	21.81 c	10.79	2.03
	CP × Control	22.47 cd	11.16	2.04
	CP × Flooding	18.48 b	11.61	1.60
	*S.1* × NP × Control	22.33 cd	10.13	2.23
	NP × Flooding	14.87 a	7.97	1.90
	CP × Control	22.97 de	9.20	2.50
	CP × Flooding	13.83 a	8.59	1.62
Genotype (1)		^∗∗∗^	^∗∗∗^	^∗∗^
Photoperiod (2)		^∗∗∗^	ns	ns
Treatment (3)		^∗∗∗^	ns	^∗∗∗^
	1 × 2	^∗^	ns	ns
	1 × 3	^∗∗∗^	ns	ns
	2 × 3	^∗∗∗^	ns	ns
	1 × 2 × 3	^∗∗^	ns	ns

*To simplify, only the results of 2016 are reported, since a similar trend in the 2-years tests was observed. Within each column, means followed by the same letters are not significantly different at P < 0.05. ^∗^P < 0.05; ^∗∗^P < 0.01; ^∗∗∗^P < 0.001.*

### Energetic Adaptation, ADH and Respiratory Activity

**FIGURE 5:**
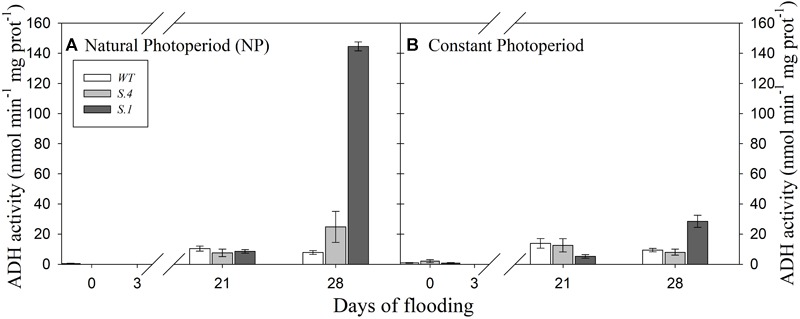
Alcohol dehydrogenase (ADH) activity detected in the roots of the genotypes during flooding under NP **(A)** and CP **(B).** Activity was determined as nmol min^-1^ mg protein^-1^. Histograms represent the mean of three biological repeats (*N* = 3), ± SD. Differences were accepted as statistically significant when *P* < 0.05.

The ADH activity of the roots was similar among the three genotypes under NP conditions, after 21 days of flooding (Figure 5A). At the CP where the photoperiod regime was like mid-July, ADH activity differed among genotypes: the roots of *WT* and *S.4* plants showed a higher ADH activity than those of *S.1* plants after 21 days of flooding (Figure 5B). These values were higher than the ones detected on the same day under NP, except for those of the *S.1* plants. On day 28, regardless of the photoperiod, ADH activity increased significantly in the roots of *S.1* plants, reaching 16-fold higher values than those detected on day 21 under NP (Figure 5A). Also, ADH activity increased more than twofold in the roots of *S.4*-plants under NP, while it remained constant or slightly decreased in *WT*-plants (Figure 5A). At the 28th day under CP conditions, *WT* and *S.4* plants retained a similar or slightly decreased of ADH activity than that determined at 21 days of root waterlogging, unlike the *S.1* genotype where it increased by 4.5-fold (Figure 5B).

**FIGURE 6:**
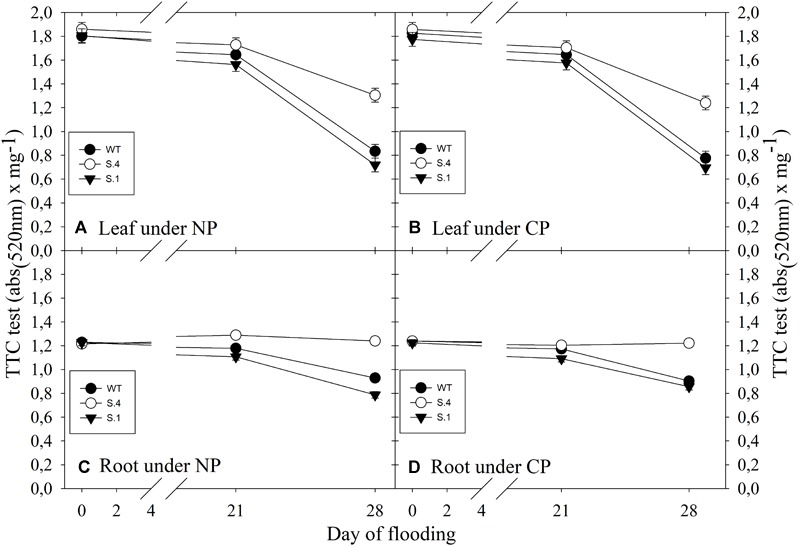
Respiratory activity (as TTC reactivity) detected in the leaf **(A,B)** and in the root **(C,D)** during flooding trials. Symbols represent the average of five biological replicate samples and bars represent ± SD. In the case of very low value of SD the bar is covered by the symbol.

The TTC (triphenyltetraziolum chloride) test was used to establish the respiratory activity in leaves and roots during flooding under the two photoperiods. The photoperiod regime did not affect the respiratory activity and any statistical difference has resulted from the ANOVA analysis in the 2 years consecutive tests, and analogous reproducible results have also been detected. In the leaves of flood-stressed plants, TTC activity decreased in all clones (Figure 6A,B). In the *S.4* plants, the decline in activity of 31% was less substantial, compared to that of the plants of the other two genotypes, where the decline was of around 56 and 60% in *WT* and *S.1* plants, respectively, for both photoperiods (Figure 6A,B). The total decrease in TTC activity detected in the roots was lower than leaves, and the decline was more significant in the *WT* and *S.1* plants, regardless of the exposure to a photoperiod regime (Figure 6C,D). The *S.4* genotype confirmed the better ability to withstand the electron transport chain compared to the other clones.

### Gas Exchange Parameters

Gas exchange parameters were detected on the 1st apical fully expanded leaves from the stem apex, throughout the flooding experiments. While the 4th healthy fully expanded leaves, of both the *S.1* and *WT* genotypes were completely dried or were greatly damaged after the 14th day of flooding (Figure 2D,G), therefore, from that moment onwards, gas exchange detection was only carried out on the 1st leaf of each genotype, and on the 4th leaf only in *S.4* plants. Variance-analysis between photoperiod, genotype and time of flooding showed no significant interaction for all the parameters of gas exchange, as detected in the 1st leaf (Table 2).

**Table 2:** Gas exchange parameters measured at 0, 7th, and 14th days from the beginning of flooding stress for plant of the three genotypes grown under NP and CP, detected during the 2016 test.

	First fully expanded leaf				
Main factors	A	*gs*	Ci	E	LWUE
Genotype *WT*		4.55 a	0.121 b	269.0 b	2.406 b	1.898
	*S.4*	5.49 b	0.099 a	239.5 a	2.076 ab	2.742
	*S.1*	4.44 a	0.098 a	249.8 ab	1.988 a	2.420
Photoperiod NP		4.77	0.112 b	261.4 b	2.259	2.225
	CP	4.89	0.096 a	244.1 a	2.051	2.482
Flooding Time 0		5.64 c	0.107	238.0 a	2.303 b	2.561
	7	4.96 b	0.110	252.7 ab	2.234 b	2.297
	14	3.89 a	0.102	267.6 b	1.919 a	2.203
Interaction					
	*WT* × NP × 0	5.60	0.140	266.7	2.803	1.998
	NP × 7	4.88	0.150	274.7	2.820	1.778
	NP × 14	3.30	0.126	297.0	2.213	1.490
	CP × 0	5.69	0.107	241.7	2.513	2.282
	CP × 7	4.92	0.102	241.7	2.870	2.165
	CP × 14	2.93	0.102	292.3	1.800	1.677
	*S.4* × NP × 0	5.64	0.082	222.0	1.750	3.230
	NP × 7	5.27	0.105	254.3	2.113	2.508
	NP × 14	4.86	0.085	230.3	1.677	3.091
	CP × 0	6.28	0.125	242.7	2.650	2.465
	CP × 7	5.60	0.097	236.7	2.250	2.497
	CP × 14	5.27	0.102	251.0	1.990	2.662
	*S.1* × NP × 0	5.06	0.120	262.0	2.423	2.160
	NP × 7	4.60	0.115	258.7	2.247	2.062
	NP × 14	3.74	0.124	287.3	2.287	1.708
	CP × 0	5.56	0.068	193.0	1.737	3.29
	CP × 7	4.49	0.091	250.3	1.690	2.771
	CP × 14	3.21	0.073	247.3	1.547	2.588
Genotype (1)		^∗∗∗^	^∗∗^	^∗∗^	^∗^	^∗∗∗^
Photoperiod (2)		ns	^∗∗∗^	^∗^	ns	ns
Flooding Time (3)		^∗∗∗^	ns	^∗∗^	^∗^	ns
	1 × 2	ns	^∗∗∗^	^∗^	^∗∗∗^	^∗∗^
	1 × 3	^∗∗^	ns	ns	ns	ns
	2 × 3	ns	ns	ns	ns	ns
	1 × 2 × 3	ns	ns	ns	ns	ns

*The factorial analysis was run on Net photosynthesis (A_*CO2*_), stomatal conductance (gs), sub-stomatal CO_*2*_ concentration (Ci), transpiration rate (E), and Leaf Water Use Efficiency (LWUE, A_*CO2*_/gs). Within each column, means followed by the same letters are not significantly different at P < 0.05. ^∗^P < 0.05; ^∗∗^P < 0.01; ^∗∗∗^P < 0.001.*

During flooding, photosynthesis (A_CO2_) detected in the 1st leaf significantly decreased on the 14 day in *WT* and *S.1* plants, in contrast to *S.4* plants (Table 2 and Supplementary Figure S1), irrespective of photoperiod. The A_CO2_ values had a 40–50% drop in the *S.1* and *WT* 1st leaf and at the 14th days of flooding, irrespective of photoperiod, whereas in *S.4* the reduction was around 14–16%. The photosynthetic capacity was also related to leaf position, resulting higher in the 4th leaf of *S.4* than in the other two genotypes at the 14th days of flooding, and was fairly constant throughout the stress, albeit the observation of a partial reduction (Table 2 and Supplementary Figure S1). Stomatal conductance (*gs*) in plants undergoing the flooding stress, detected in the leaves of both *S.1* and *WT* plants was higher in NP than CP (Table 2 and Supplementary Figure S2). In *S.4* plants, on the other hand, *gs* values, overall, resulted generally higher for those under CP. However, the values were lower than those detected in leaves of the other two genotypes, thus indicating a partial closure of the stomata (Table 2). Only the interaction between Genotype and Photoperiod was statistically significant (Table 2 and Supplementary Materials). Sub-stomatal CO_2_ concentration (Ci), measured in the 1st leaf under flooding stress, increased at the 14th day irrespective of genotype (Table 2 and Supplementary Figure S3), whereas it decreased under CP reaching its lowest value in *S.4*-plants. A significant interaction between Genotype and Photoperiod resulted from the ANOVA test (Table 2), pointing out that under CP Ci was lower in *WT* and *S.1* plants than under NP (Supplementary Figure S3). The 4th leaves of the *S.1* and *WT* plants showed a similar behavior to Ci, i.e., it slightly increased during flooding, while in the *S.4* leaves it retained similar values on both photoperiodic conditions and during the flooding (Supplementary Figure S3). The leaf transpiration rate (E) decreased under flooding stress, and although photoperiod was not significant, the lowest values were detected in *S.1* plants under CP (Table 2). ANOVA highlighted an interaction between Genotype and Photoperiod significantly higher (Table 2 and Supplementary Figure S4) in *S.1* and *WT* plants and was like LWUE which, during flooding stress, decreased more under NP than under CP, whereas it kept generally stable in *S.4* plants (Table 2 and Supplementary Figure S5). The *S.4* 4th leaves behaved like the one of the other genotypes, while the 1st leaves maintained a significant value of LWUE. This can be explained by an active metabolism still working during stress.

### Soluble Sugars

There was a statistically significant interaction between the main factors (Genotype, Photoperiod, and Flooding) for glucose, fructose, and sucrose detected in the leaves, whereas no significant interaction was shown for the accumulation of sorbitol (Table 3A). The behavior of each genotype was similar when exposed to the two photoperiodic regimes under normoxic condition, the highest amount of sugars was detected in leaves of plants grown under NP, except for sucrose and sorbitol detected in the leaves of *WT*-plants. In contrast, the photoperiod regime has a not negligible effects in plants exposed to flooding stress (Table 3A).

**Table 3:** (A) Carbohydrates detected in leaves of *S.1, S.4*, and *WT* plants, exposed to natural photoperiod (NP) and constant photoperiod (CP) after 14 days of flooding.

Main factors		Leaves			
		Glucose	Fructose	Sucrose	Sorbitol
Genotype	*WT*	0.919 b	0.445 b	2.201 b	2.329 a
	*S.4*	0.215 a	0.144 a	1.527 ab	2.076 a
	*S.1*	1.131 c	0.862 c	1.114 a	3.673 b
Photoperiod	NP	1.077 b	0.685 b	1.874	3.080 b
	CP	0.433 a	0.282 a	1.354	2.306 a
Treatment	Control	0.580 a	0.345 a	1.513	2.607
	Flooding	0.930 b	0.622 b	1.716	2.779
Interaction					
	*WT* × NP × Control	0.901 bc	0.339 b	1.338 a	2.226
	NP × Flooding	1.653 d	0.985 d	3.301 c	2.401
	CP × Control	0.354 ab	0.195 ab	2.869 c	2.523
	CP × Flooding	0.768 b	0.262 ab	1.298 a	2.167
	*S.4* × NP × Control	0.314 a	0.174 ab	1.667 b	1.975
	NP × Flooding	0.194 a	0.169 ab	1.436 ab	2.470
	CP × Control	0.192 a	0.099 a	1.479 ab	1.802
	CP × Flooding	0.161 a	0.135 a	1.527 ab	2.058
	*S.1* × NP × Control	1.123 c	0.692 c	1.156 a	4.303
	NP × Flooding	2.277 e	1.755 e	2.349 bc	5.107
	CP × Control	0.597 b	0.574 c	0.567 a	2.813
	CP × Flooding	0.528 ab	0.427 bc	0.384 a	2.472
Genotype (1)		^∗∗∗^	^∗∗∗^	^∗^	^∗∗∗^
Photoperiod (2)		^∗∗∗^	^∗∗∗^	Ns	^∗∗∗^
Treatment (3)		^∗∗∗^	^∗∗∗^	Ns	ns
	1 × 2	^∗∗∗^	^∗∗∗^	Ns	^∗∗∗^
	1 × 3	^∗∗∗^	^∗∗∗^	Ns	ns
	2 x3	^∗∗∗^	^∗∗∗^	^∗∗^	ns
	1 × 2 x3	^∗∗∗^	^∗∗∗^	^∗^	ns

*Values represent the average of each carbohydrate, expressed as mg for gram of dry weight, as detected in three biological samples. Different letters indicate statistically difference among the column of each factor and interaction. Within each column, means followed by the same letters are not significantly different at P < 0.05. ^∗^P < 0.05; ^∗∗^P < 0.01; ^∗∗∗^P < 0.001.*

Irrespective of the genotype, the quantity of the carbohydrates analyzed was basically higher in leaves of CP plants subjected to flooding (Table 3). Noteworthy, the amount of glucose and fructose in the *S.1* leaves, under normoxic conditions, was higher in NP than CP, and this sugars accumulation behavior during the exposure to flooding stress was increased (Table 3A). An analogous trend was observed for the quantity of carbohydrates detected in *WT* leaves, except for sucrose and sorbitol; in fact, in flooded plants exposed to CP the amount of the sugars decreased (Table 3A). In the leaves of *S.4* plants, the amount of glucose, fructose, and sorbitol did not statistically differed, instead, irrespective of the photoperiod regime the amount of sorbitol increased under flooding stress (Table 3A).

The factorial analysis shows a statistically significant interaction between the main factors (Genotype, Photoperiod, and Flooding) for the accumulation of sucrose and sorbitol in root tissues, while no significant interaction resulted from glucose and fructose accumulation (Table 3B). In the *S.4*-roots a major accumulation of all analyzed carbohydrates was detected, differently from the other genotypes (Table 3B). Under normoxic conditions, the accumulation of carbohydrates was highest in the *S.4*-root grown under NP; conversely, under anoxic conditions the accumulation of carbohydrates resulted highest in CP roots, except for glucose. Even in *WT* roots, under normoxic conditions, the accumulation of carbohydrates resulted highest in the roots of plant grown under NP, except for sucrose (although not significant) (Table 3B). In the roots of flooded-plants the accumulation of carbohydrates was markedly reduced in CP plants, where the values were halved, except for sorbitol. In the *S.1*-roots there were detected the lowest values of carbohydrate-accumulation, irrespective of photoperiodic and stress conditions (Table 3B).

**Table 3:** (B) Carbohydrates detected in roots (b) of *S.1, S.4*, and *WT* plants, exposed to natural photoperiod (NP) and constant photoperiod (CP) after 14 days of flooding.

Main factors		Roots			
		Glucose	Fructose	Sucrose	Sorbitol
Genotype	*WT*	6.920 b	4.173 b	6.586 b	6.033 b
	*S.4*	7.097 b	5.590 a	10.020 c	7.170 c
	*S.1*	1.430 a	1.538 b	1.829 a	2.007 a
Photoperiod	NP	6.249 b	4.122 b	6.048	5.410 b
	CP	4.049 a	3.412 a	6.243	4.730 a
Treatment	Control	6.245 b	4.346 b	6.367	5.708 b
	Flooding	4.053 a	3.188 a	5.924	4.432 a
Interaction				
	*WT* × NP × Control	10.610	5.659	5.823 bc	7.680 f
	NP × Flooding	8.336	4.661	8.562 d	5.47 cd
	CP × Control	5.720	4.036	7.539 cd	7.205 ef
	CP × Flooding	3.017	2.335	4.420 b	4.199 c
	S.4 × NP × Control	10.300	6.626	12.050 e	8.937 g
	NP × Flooding	5.320	4.671	6.098 c	6.363 e
	CP × Control	8.055	6.031	8.409 d	5.518 de
	CP × Flooding	4.175	5.032	13.530 f	7.864 fg
	S.1 × NP × Control	1.639	1.984	2.363a	2.724 b
	NP × Flooding	1.293	1.130	1.398 a	1.709 g
	CP × Control	1.149	1.740	2.024 a	2.187 fg
	CP × Flooding	1.641	1.299	1.531 a	1.408 g
Genotype (1)		^∗∗∗^	^∗∗∗^	^∗∗∗^	^∗∗∗^
Photoperiod (2)		^∗∗∗^	^∗∗^	ns	^∗∗^
Treatment (3)		^∗∗∗^	^∗∗∗^	ns	^∗∗∗^
	1 × 2	^∗∗∗^	^∗∗^	^∗∗∗^	ns
	1 × 3	^∗∗∗^	ns	ns	^∗∗∗∗^
	2 × 3	ns	ns	^∗∗^	^∗∗^
	1 × 2 × 3	ns	ns	^∗∗∗^	^∗∗∗^

*Values represent the average of each carbohydrate, expressed as mg for gram of dry weight, as detected in three biological samples. Different letters indicate statistically difference among the column of each factor and interaction. ^∗^P < 0.05; ^∗∗^P < 0.01; ^∗∗∗^P < 0.001.*

### Principal Component Analysis

Principal component analysis carried out on the data for all the analyzed set of parameters identified two synthetic variables, that explain 64.2% of the variability, with component1 (PC1) accounting for 51.0%, and component2 (PC2) for 13.2%. The PCA scatter-plot split the samples into three main groups. The position of the samples summarizes the phenotypic variability of *Prunus* genotypes in the response to Flooding stress, which is the main factor that affect the separation on PC1 (Supplementary Figure S6A). The separation induced by this factor didn’t occur at the same extent inside of each genotype. All *S.4*-plant samples were grouped in the lower right quadrant, characterized by the improved tolerance, photosynthetic performance even at the 4th leaf, and high energy activity in leaf and root. Inside the other two genotypes the PC1 synthetic variable contributed to a strong clustering of the normoxic plants from the anoxic plants. The photoperiod factor contributed to separation on PC2 (Supplementary Figure S6A), with different extent among the genotypes, high pronounced in *S.1* genotype, less pronounced in *WT* genotype and almost absent in *S.4* genotype.

An exhaustive view of the morphological, physiological and carbohydrates accumulation and partitioning in leaf and root of plants of the three genotypes in response to flooding stress under photoperiodic conditioning was obtained through principal component analysis (PCA), as reported in Supplementary Figure 6B. PC1 was positively correlated to CHLA, CHL-tot, FW, and DW leaf area, TTC leaf and Root, Drop-leaf, and many of the photosynthetic parameters of the 1st and the 4th leaf. PC1 was also negatively correlated to glucose (GL) and fructose (FR) accumulation in the leaf. PC2 was positively correlated to GL-Root, SU-leaf, GL-leaf, and FR-leaf, CHL-ratio, gs-leaf1, Ci-leaf1, and E-leaf1. Moreover, PC2 was negatively correlated with WUE-leaf1. The most informative variables (loadings > | 0.3|) were leaf gas exchange parameters, followed by drop leaf, TTC activity and chlorophylls content. Among the carbohydrates glucose and fructose resulted informative, suggesting that the gas exchange, energy activity and glucose and fructose accumulations are more involved in adaption to a waterlogged environment of the *S.4* genotypes (Supplementary Figure 6B).

## Discussion

Investigation on rainfall variations has been carried out several times in both Northern and Southern hemispheres, focusing on the Mediterranean environments, and considering different parameters such as total precipitations, intensity, temporal repetition, etc. (Alpert et al., 2002; Homar et al., 2010; Trenberth, 2011; Arnone et al., 2013; IPCC, 2014). The extent and the variation of extreme environmental changes affect the functioning of agroecosystems. Crop production, food security and stability depend on the impact of these changes on the previous conditions and on the plasticity of crop plants to adapt to new environmental scenarios. Therefore, the central goal of the agricultural industry is to reach the security and stability of food available for human-consumption (Dhankher and Foyer, 2018).

In temperate and higher latitude countries, persistent flooding usually occurs in autumn, concomitantly with the physiological and biochemical events triggered by vernalization process which could also have evolved for plant survival against anoxic soil condition. To cope with the seasonal change of environmental conditions, perennial woody plants have evolved a “memory” to keep themselves synchronized and activating appropriate adaptive strategies. Photoperiod is one of the main seasonal cues perceived by plant that indicate the change of seasons in each location. Photoperiodic signals regulate the synchrony or asynchrony of plant phenology and the adaptation to the functionally related environmental cues (Kobayashi and Weigel, 2007; Hänninen and Tanino, 2011).

In this study, the possible connections between the photoperiodic signal perceived by plants and the adaptive response to flooding stress have been explored in somaclonal variants of *P. cerasifera* with divergent ability to tolerate waterlogging. *Prunus* are considered a flood-sensitive species (Domingo et al., 2002; Amador et al., 2012; Almada et al., 2013; Rubio-Cabetas et al., 2018) because few days of hypoxia and/or anoxia lead to a strong reduction in the growth and production, compromising plant survival under prolonged exposure. Other than *WT* genotype, two previously characterized somaclonal mutants were included in the experimental design: *S.1* and *S.4* having a lower and higher tolerance compared to *WT*, respectively (Pistelli et al., 2012; Iacona et al., 2013).

The extension of daylight hours number (constant photoperiod, CP) was able to block the signal induced by the seasonal changes, allowing to extend the growth period in late summer and autumn, when *Prunus* spp. usually arrest aerial growth. As ascertained by gas exchanges measures, light intensity was below the compensation point but efficient to maintain long day photoperiodic signal. Under CP conditions, plants of the three clones escape growth cessation and continued to differentiate new phytomers compared to those exposed to normal photoperiod (NP). The longer the waterlogging period, the higher the sensitivity to anoxic conditions in the *WT* and *S.1* genotypes grown in CP, as supported by the higher percentage of leaves abscission and desiccation. In contrast, the photoperiod modification only slightly affects the flooding-tolerant *S.4* clone, which showed an increase of leaves abscission only after a prolonged period of exposure to waterlogging conditions. Flooded *S.4* plants continued to grow, developing new shoots and suckers as well as roots from stems, that increased root conductivity, as it was previously reported (Pistelli et al., 2012). These features indicated that the *S.4* genotype has acquired a tolerance to flooding stress regardless of the photoperiod. In previous experiments performed during July, whole plants generated from a graft combination between cv Suncrest peach and *S.4* (rootstock) underwent 21 days of flooding, and strong tolerance to anoxia resulted compared to the *WT* and minus variant genotype *S.1* (Iacona et al., 2013). In our study, *WT* and *S.1* plants under NP showed symptoms of stress later than the plants under CP, as flooding persisted. Essentially, leaves undergo edge necrosis, wilt and abscission very rapidly under CP, i.e., plants were not able to activate any efficient strategy to counteract anoxia. Conversely, plants were somehow able to oppose the damages induced by anoxia under NP. These results may suggest a decreasing plants ability of adapting to stress occurring in an asynchronous period (e.g., during summer), while they are potentially capable of responding to stress during a synchronous period (late summer and/or fall) (Pistelli et al., 2012; Iacona et al., 2013).

Apart from morphological evidences, other metabolic and physiological parameters were analyzed, in order to collect more information about adaptation mechanisms and photoperiod effects. A_CO2_ of leaves was not affected by the photoperiod, although the interaction between genotypes and time of waterlogging resulted significant. The decline in A_CO2_ of flooded-leaves was observed in both sensitive and tolerant genotypes and was clearly associated with the increase in Ci in both the *S.1* and *WT* genotype. The increase in Ci due to the reduction in A_CO2_ causes stomatal closure, with a consequent decrease in gs, in agreement with previous reports (Pérez-Jiménez et al., 2017, 2018). The increase of Ci concomitant with a decrease in chlorophyll in *S.1* and *WT* plants compared to normoxic plants, indicates that non-stomatal factors may play a prevalent role in the limitation of A_CO2_ compared to stomatal conductance. In citrus, the limitation of A_CO2_ in flood-stressed plants was not associated to non-stomatal factors, such as chlorophyll degradation and chlorophyll fluorescence, that were more determinant than stomatal limitation on A_CO2_ (García-Sánchez et al., 2007). The leaves of the *S.4* flooded-plants retained their functions under stress conditions, indicating that this genotype allowed an active metabolism with a good photosynthetic activity. The observed slight reduction in A_CO2_ detected in flooded-leaves was not accompanied by any significant variation in Ci, and *gs* remained largely constant. Moreover, no difference in gas exchange parameters was detected under the two photoperiods.

Total chlorophylls content resulted lower in all genotypes under CP. The decrease of chlorophyll *a* in *S.4* plants is less prominent than in the other genotypes, especially under NP. Moreover, a significant interaction was detected for its content among photoperiod, genotype and flooding. Instead, chlorophyll *b* increased in *S.4* flooded leaves and decreased in the corresponding leaves of *WT* and *S.1*. These data indicate that the photosystems were better preserved in *S.4* genotype as it is reported for tolerant species, counteracting the decrease in the efficiency of the harvested light energy generated by the damage in the photosystems (Mauchamp and Méthy, 2004; Gururani et al., 2015). This justifies the higher capability of the *S.4* leaves to retain a photosynthetic activity even in the 4th leaf (Supplementary Material) under a condition of anoxia, regardless of photoperiod. Essentially, under anoxic flooding stress the inhibition of photosynthesis can take place by either a reduction in the photosynthetic activity due to protective mechanisms, or by photoinhibition (Mauchamp and Méthy, 2004; Fernández, 2006).

The *S.4* plants under anoxic conditions, despite photoperiod, kept a sufficiently efficient metabolism throughout the flooding period, and although it may be reduced after 28 days of stress, they produced energy and maintained a higher respiratory activity than the other two genotypes both in the leaves and in the roots. Although carbohydrate-availability in the leaves and in the roots of the *S.4* flooded-plants was reduced, the total chlorophyll content underwent a slight reduction, however, the TTC activity never dropped below 60% of the initial value. The high TTC levels observed in the roots can be attributed to the slight loss in the oxygen supply, since active metabolism might still have occurred.

An efficient use of carbohydrates and their accumulation within plant organs especially to the root tissues is indeed a crucial factor for preserving cell functionality during flooding stress (Parent et al., 2008). The maintenance of carbohydrate reserves and capacity to metabolize them to sustain ATP levels at anoxic conditions is preserved in flood-tolerant tree, along with the avoidance of toxic compounds accumulation that could damage the membrane integrity (Kozlowski and Pallardy, 2002; Parent et al., 2008). The sugar content showed a significant difference among the genotypes. Independently from photoperiod, *S.4* showed a functional leaf with a preserved photosynthetic activity, and the lower levels of sugars, except for sorbitol, are probably linked to the capacity to translocate the sugars from the leaves to the roots (Noiraud et al., 2001; Centritto, 2005). The higher content of sorbitol detected in leaves of flooding-exposed plants could be associated to the double role of sorbitol, as main load transported sugar, e.g., in the roots, and as an agent for the osmoregulation of the leaf under abiotic stress (Lo Bianco et al., 2000; Centritto, 2005). In a previous work, a high level of expression of sorbitol transporter-1 gene (*SOT1*) had been detected in both leaf and root tissues in plants subjected to anoxia (Pistelli et al., 2012), indicating an active role in translocation of this sugar to counteract the stress. It is recognized that plants under prolonged flooding are subjected to starvation and that the leaves retain non-structural carbohydrates to hinder the progress of oxidative stress, therefore they are accumulated in the tissues and not translocated (Tamang and Fukao, 2015). The sugar-content in the leaves of the *WT* and *S.1* genotypes confirm this scenario in flooded-plants under NP. Under CP, higher amounts of sugars in the anoxic-leaves were found only in the *WT*-genotype, while in the anoxic-leaves of the *S.1*-genotype, the amount was equal to or lower than normoxic leaves, even for sorbitol. These results confirm once again that the *S.1* genotype is very anoxic-sensitive, and this behavior is worsened by an extended photoperiod. The reduced amount of sugars found in the anoxic-roots of the *S.1* genotype compared to that of the other two genotypes, indicates that under prolonged flooding conditions the *S.1* roots undergo to degeneration of the tissues, as it was previously reported (Pistelli et al., 2012).

It is understood that the anaerobic condition of roots alters plant growth and respiration, and ADH activity plays a key role in producing ATP (Geigenberger, 2003; Kreuzwieser et al., 2004). ADH activity in *WT* and *S.4* genotypes showed a similar behavior with perpetuated flooding under both NP and CP, confirming the previous study performed during early summer (Pistelli et al., 2012). If during the summer period (and/or CP) this increase can be linked to a tolerance, already expressed at the beginning of flooding (Toro et al., 2018), under NP this behavior could be attributed to the seasonal change linked to the process of vernalization, i.e., plants subject to vernalization could acquire a tolerance to flooding. On the other hand, *S.1* plants showed different and very high level of ADH with the prolonged flooding, more prominent in NP rather than in CP. This big increase could be due to the leakage in the roots, and great fermentation processes, and it is supported by the recent observation on other root respiration components of the *Prunus* rootstock (Toro et al., 2018), where long term flooding tolerance depends on the responses induced in the short term hypoxia: *S.1* is always sensitive to flooding, confirming its minus variant status (Pistelli et al., 2012).

In this paper we have focused our attention on carbohydrates, although other compounds play a role in the regulation of abiotic stress and can be involved in the flooding stress. Protective molecules as proline, but also glycine-betaine, can be produced and accumulated for the osmotic adjustment during salinity and/or drought stress, as well as GABA shunt can contribute to the dissipation of energy excess and CO_2_ release, and support the electron transport chain where ROS could increase (Carillo, 2018; Annunziata et al., 2019). Further research on the involvement of these compounds in the flooding tolerance could lead to an improvement of the knowledge of this mechanism. Furthermore, the redox state is also important to maintain a balance between energy production and consumption, and during several abiotic stress this equilibrium is affected by the production of oxidative compounds (Suzuki et al., 2012). Reactive oxygen species (ROS) are common compounds of several abiotic stress, leading to damage of membrane integrity and alteration of chloroplast electron chain (Suzuki et al., 2012). The production of secondary metabolites, as antioxidant compounds (polyphenols and others) are considered good scavenging products to remove ROS. In our previous paper (Pistelli et al., 2012) these secondary metabolites were determined, and their contribution of flooding tolerance has been already discussed.

Recent studies (Alfieri et al., 2015) aimed at evaluating climate projections using the EURO CORDEX RCP8.5 scenarios, show a positive trend for the maximum daily precipitations in most European countries by the end of the century. Furthermore, a significant increase in the frequency of extreme events larger than 100% is predicted for the period 2006-2035 in 21 out of 37 European countries with a worsening in the following period until 2095. Changes in intensity and persistence of flooding and drought occur significantly during the summer all over the European region, with Central Mediterranean and Central/Western Europe particularly vulnerable to these phenomenon (Pal et al., 2004). The moisture holding capacity of the atmosphere due to the climate change, in fact, can increase the strength of the precipitation and the occurrence of flood events (Trenberth et al., 2003).

Phenotypical plasticity is required to counteract the new occurring scenario where extreme daily rainfall will become frequent. Therefore, rather than resistant crop plants the new agronomic contest require plants with an evolved plasticity that shift from submergence to the de-submergence without undergoing physiological stress. This happens only if plants will be not constrained by the regulatory cross-talk related with daily-length seasonal change (photoperiod).

To our knowledge, this is the first report addressing the relationship between photoperiod (seasonal changes) and adaptive plant response to flooding. Results indicate that *S.4* genotype is tolerant to flooding regardless of photoperiodic regime, while *S.1* and *WT* genotypes are less sensitive or tolerant under NP respect to CP, when natural daylight becomes shorter (September-October) and vernalization process has been already started. Although an interaction with photoperiod appear evident at morphological level, metabolic and physiological analyses does not depict a clear framework. The molecular and physiological cross-talk that may exist between these two plant biological processes should not be considered unreal, since recent papers have shown that photoperiod and circadian rhythm play a key role in the adaptive responses to stresses in *Arabidopsis* and in some trees (Artlip et al., 2013; Grundy et al., 2015; Lloret et al., 2018). This cross-talk must be completely explored, and the *S.4* and *S.1* genotypes studied, that share an identical genetic background with the *WT*, provide an interesting study material to contribute for deciphering the signaling elements of the regulatory networks governing the dialogue itself. The *S.4* genotype has an increased tolerance that appears possibly to be independent from photoperiod, while the *S.1* genotype has an increased susceptibility to anoxia. A comprehensive understanding on how adaptive responses to flooding stress are linked to photoperiod is pre-requisite for the development of adapted genotypes to the environmental anoxic fluctuations. Furthermore, understanding the regulation of the adaptation to extreme flooding conditions under the climate changes may suggest cultural practices to ameliorate their negative impact.

## Author Contributions

RMu, CI, and LP developed the concept and with RMa and MR wrote the manuscript. CI, MC, and LG performed eco-physiological analysis, collected, and analyzed meteorological data. LP performed sugar and ADH analysis and quantification. RMa and MR discussed climate changes. LG, MC, and RMa performed statistical analyses. All authors discussed and commented on the manuscript.

## Conflict of Interest Statement

The authors declare that the research was conducted in the absence of any commercial or financial relationships that could be construed as a potential conflict of interest.

## Abbreviations

A_CO2_: net CO_2_ assimilation rate
Ci: intercellular CO_2_ concentration
CP: constant photoperiod
E: leaf transpiration rate
*gs*: stomatal conductance
LWUE: leaf water-use efficiency
NP: natural photoperiod
