# Supplementary material for: Day-Length Is Involved in Flooding Tolerance Response in Wild Type and Variant Genotypes of Rootstock Prunus cerasifera L
Source: Front Plant Sci. 2019 May 3;10:546. doi: 10.3389/fpls.2019.00546 (PMC6509233; doi:10.3389/fpls.2019.00546)
Supplement: Supplementary file 1 [file Table_1.docx]

**Supplementary Material**

**Day-length is involved in flooding tolerance response in wild type and variant genotypes of rootstock *Prunus cerasifera* L.**

**Calogero Iacona^1,*^, Laura Pistelli^1,*^, Marco Cirilli^2,3^, Lorenzo Gatti^2^, Roberto Mancinelli^2^, Maria Nicolina Ripa^2^ and Rosario Muleo^2+^**

^1^Department of Agriculture, Food and Environment, University of Pisa, Pisa, Italy

^2^Laboratory of Molecular Ecophysiology of Woody Plant, Department of Agricultural and Forestry Sciences, University of Tuscia, Viterbo, Italy;

^3^Department of Agricultural and Environmental Sciences, University of Milan, Milan, Italy

**Supplementary Figure S.1:** Effects of flooding and photoperiod on the photosynthetic assimilation (A_CO2_) in leaves of plum plants rootstock Mr.S. 2/5-*WT* (a, b), and its *S.1* (c, d) and *S.4* (e, f) variant genotypes. The A_CO2_ values were detected at 1^st^ and 4^th^ leaves, excepted for *WT* and *S.1* genotypes at Day 14 after flooding. Values are means ± SD of five biological replications.

**Supplementary Figure S.2:** Effects of flooding and photoperiod on the stomatal conductance (*gs*) in leaves of plum plants rootstock Mr.S. 2/5-*WT* (a, b), and its *S.1* (c, d) and *S.4* (e, f) variant genotypes. The *gs* values were detected at 1^st^ and 4^th^ leaf, excepted for *WT* and *S.1* genotypes at Day 14 after flooding. Values are means ± SD of five biological replications.

**Supplementary Figure S.3:** Effects of flooding and photoperiod on the internal CO2 concentration (Ci) in leaves of plum plants rootstock Mr.S. 2/5-*WT* (a, b), and its *S.1* (c, d) and *S.4* (e, f) variant genotypes. The Ci values were detected at 1^st^ and 4^th^ leaf, excepted for *WT* and *S.1* genotypes at Day 14 after flooding. Values are means ± SD of five biological replications.

**Supplementary Figure S.4:** Effects of flooding and photoperiod on the transpiration (E) in leaves of plum plants rootstock Mr.S. 2/5-*WT* (a, b), and its *S.1* (c, d) and *S.4* (e, f) variant genotypes. The E values were detected at 1^st^ and 4^th^ leaf, excepted for *WT* and *S.1* genotypes at Day 14 after flooding. Values are means ± SD of five biological replications.

**Supplementary Figure S.5:** Effects of flooding and photoperiod on the Leaf water use efficiency (LWUE) in leaves of plum plants rootstock Mr.S. 2/5-*WT* (a, b), and its *S.1* (c, d) and *S.4* (e, f) variant genotypes. The LWUE values were calculated for the 1^st^ and 4^th^ leaf, excepted for *WT* and *S.1* genotypes at Day 14 after flooding. Values are means ± SD of five biological replications.


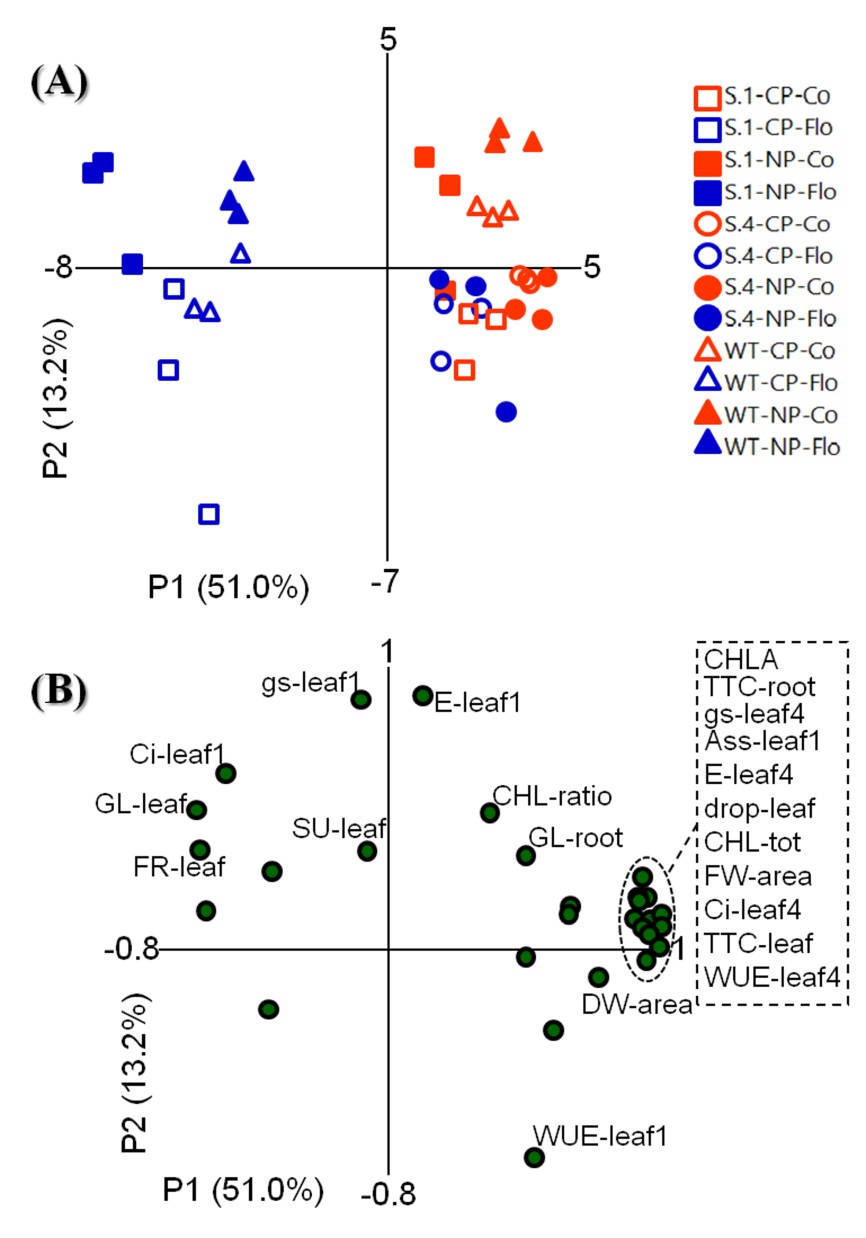


**Supplementary Figure S.6**: Multigroup discriminant analysis (MDA, A) of plants of WT, S.4 and S.1 Prunus cerasifera genotypes grown under natural photoperiod (NP) and constant photoperiod (CP), both exposed to anoxic (Flo) and normoxic (Co) conditions. Principal component analysis (PCA, B) on morphological, physiological parameters and carbohydrate concentrations of the three genotypes, in the figure only the mains contributors are highlighted. **Ass-leaf1**: net CO_2_ assimilation of the 1^st^ leaf (apical leaf); **CHLA**: chlorophyll *a*; **CHL-ratio**: chlorophyll *a*/*b* ratio; **CHL-tot**: total amount of chlorophylls; **Ci-leaf1**: intercellular CO_2_ concentration of the 1^st^ ^l^ leaf; **Ci-leaf4**: intercellular CO_2_ concentration of the 4^th^ leaf; **drop-leaf**: percentage of dropped leaves; **DW-area**: dry weight per unit of leaf area; **E-leaf1**: transpiration rate of the 1^st^ leaf; **E-leaf4**: transpiration rate of the 4^th^ leaf; **FR-leaf**: leaf fructose concentration; **FW-area**: fresh weight per unit of leaf area; **GL-leaf**: leaf glucose concentration; **GL-root**: root glucose concentration; **gs-leaf1**: stomatal conductance of the 1^st^ leaf; **gs-leaf4**: stomatal conductance of the 4^th^ leaf; **SU-leaf**: leaf sucrose concentration; **TTC-leaf**: TTC reactivity test of leaf; **TTC-root**: TTC reactivity test of root; **WUE-leaf1**: water use efficiency of the 1^st^ leaf; WUE-leaf4: water use efficiency of the 4^th^ leaf.
